# A conformal beam splitter with polarization transformation operation

**DOI:** 10.1038/s41598-023-48306-y

**Published:** 2023-12-08

**Authors:** Fahad Ahmed, Hattan Abutarboush, Naveed Ashraf, Tayeb A. Denidni, Farooq A. Tahir

**Affiliations:** 1 National Institute for Scientific Research (INRS), University du Quebec, Montréal, Canada; 2 College of Engineering, Taibah University, Madinah, Saudi Arabia; 3 Department of Electrical Engineering, The University of Lahore, Lahore, Pakistan; 4 School of Electrical Engineering and Computer Science (SEECS), National University of Sciences and Technology (NUST), Islamabad, Pakistan

**Keywords:** Engineering, Electrical and electronic engineering

## Abstract

A multifunctional beam splitting frequency selective surface (FSS) is modeled, analyzed, and tested in transmission and reflection modes. The proposed FSS comprises a C-shaped split-ring resonator designed and fabricated on an ultrathin, flexible polyimide material. When a linearly polarized incident wave interacts with the unit cell of the proposed FSS, half of the wave is reflected, and the other half is transmitted at two frequency bands from 5.8–6.2 GHz and 18.5–22 GHz. Moreover, the proposed FSS is angularly stable upto 40^°^ and also performs simultaneous beam splitting and quarter-wave operation within one of its two bands of operation i.e., from 16.5–18.2 GHz. Such flexible beam splitting FSSs with polarization transformation operation and having angular stability, size miniaturization and multi-band operation is a specialized component having potential to be used for electromagnetic wave manipulation in antenna systems, radar technology, stealth technology, wireless communication, satellite communication, medical imaging, security and surveillance, aerospace and defense, and automotive radar.

## Introduction

Beam splitter is a device that splits a beam of light into two or more separate beams. It reflects a portion of the incoming light and transmits the remainder. These devices are widely studied due to their applications in interferometers, spectrometers, quantum optics, and optical communication^1–5^. The conventional beam splitters are handicapped due to their bulky size, low beam splitter freedom, poor efficiency, and incompetency for integration on a large scale. Therefore, various metasurface/FSS-based beam splitters have been reported in the literature.

An ultrathin metasurface-based power splitter for co-polarized light in the visible frequency regime has been proposed in^6^. Li et al. have designed a metasurface using the Fourier transform technique with a vector iterative algorithm to perform the power beam splitting operation^7^. Wei et al. have reported a broadband metasurface, performing power beam splitting operations in the terahertz regime^8^. However, these metasurfaces/FSSs are able to perform only power beam splitting operations and cannot exploit the polarization state of the incident wave. Thus, a design with a quarter-wave plate operation (QWP, i.e., when a linearly polarized wave is incident on the structure, it splits the wave by changing its polarization to circularly polarized light or vice versa) is reported in the literature^9^. A silicon-based metasurface beam splitter has been reported that can split the incident wave in transmission mode^10^. Similarly, Mao et al. have proposed a high-efficiency ultra-thin beam splitter working in the frequency range of 11.65–11.95 GHz^11^. Liu et al. have designed a gauge field metamaterial offering polarization beam splitting at 12.5 GHz frequency^12^. Wan et al. have proposed a huygen metasurface performing beam splitting operations in a transmission mode at 9 GHz^13^. A reflective beam-splitting programmable surface has been reported in^14^, splitting the beam at 4.2 GHz. Phon et al. have proposed a self-deformable beam splitter operating in a reflection mode at 9 GHz^15^. Recently, another two structures have been proposed to achieve beam splitting in the THz and GHz frequency regimes, respectively^16,17^. However, all the reported designs are either power beam splitters or polarization-converting beam splitters. A multifunctional metasurface/FSS that can offer both operations, i.e., power splitting and polarization-converting beam splitting, through a single ultrathin conformal design is yet to be explored.

In this paper, we present a novel single-layer conformal beam splitter FSS that can achieve beam splitting operation (i.e., half the power is reflected and half of the power is transmitted) without any polarization transformation at two frequency bands of 5.8–6.2 GHz and 18.5–22 GHz and also polarization-converting beam splitting operation (i.e., half of the power is reflected as a left-handed circularly polarized wave, and half of the power is transmitted as a right-handed circularly polarized wave) in the frequency band of 16.5–18.2 GHz. The conformal beam splitting FSSs more efficiently use space and have excellent integration with complex surfaces, such as curved or irregularly shaped objects. It is important to analyze the physical mechanism for the QWP, or linear to circular polarization transformation, at this frequency band.

## The proposed FSS

### Geometrical configuration and the design evolution

**Figure 1:**
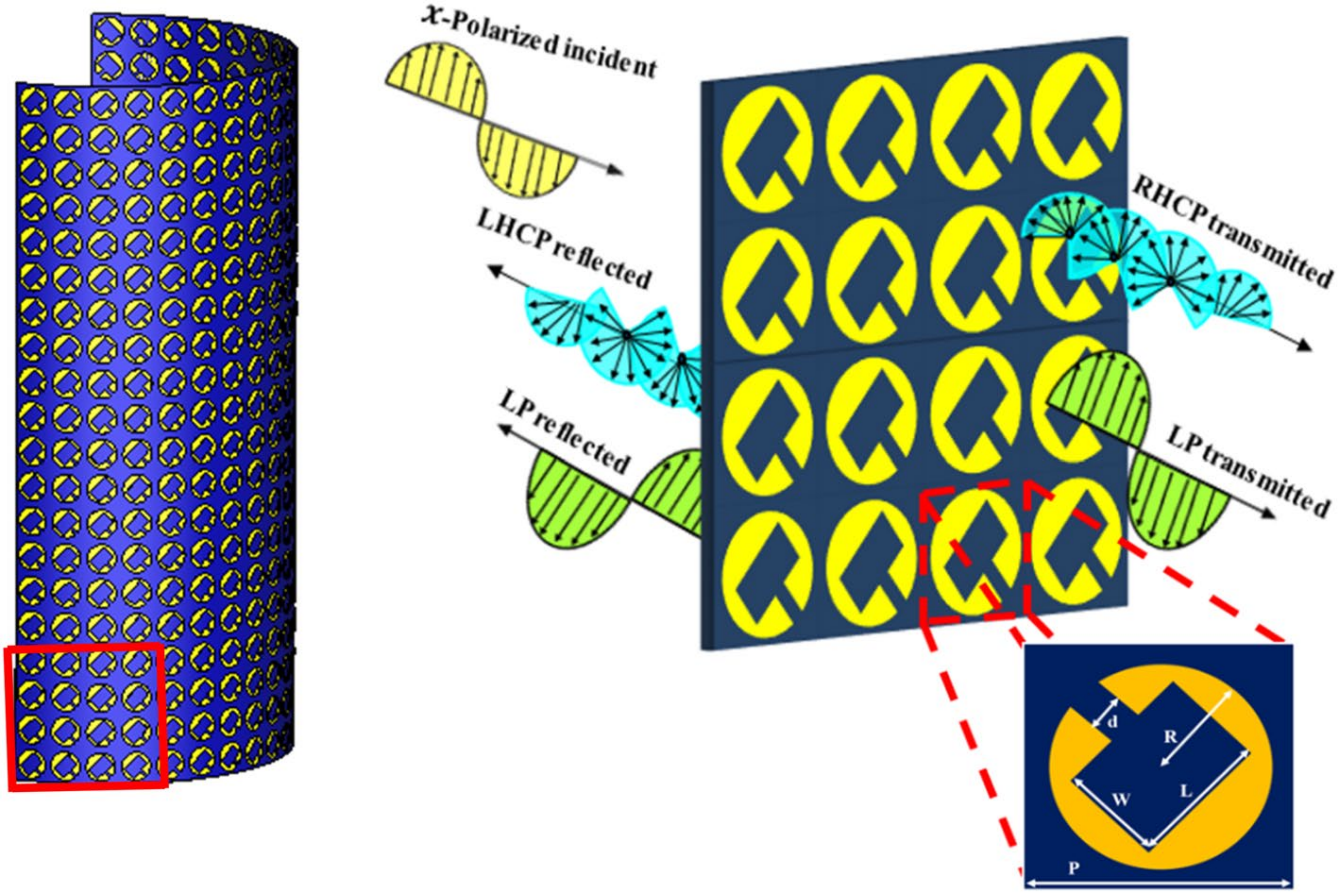
Schematic diagram of the proposed FSS.

The proposed FSS, shown in Fig. 1, is composed of a two-dimensional periodic arrangement of the unit cells along with the x- and y-directions. A C-shaped copper patch is printed on a polyimide substrate. The relative permittivity of the substrate is 3.5, and the loss tangent is 0.0027, whereas the copper conductivity is 5.8 × 10^7^S/m. The optimized physical dimensions of the structure are d = 1 mm, R = 3.5 mm, L = 6.5 mm, and W = 4 mm. The periodicity of the unit cell is p = 11 mm to ensure its operation at a lower frequency band too. The thickness of the substrate is 0.06 mm, which makes this FSS a conformal FSS.

**Figure 2:**
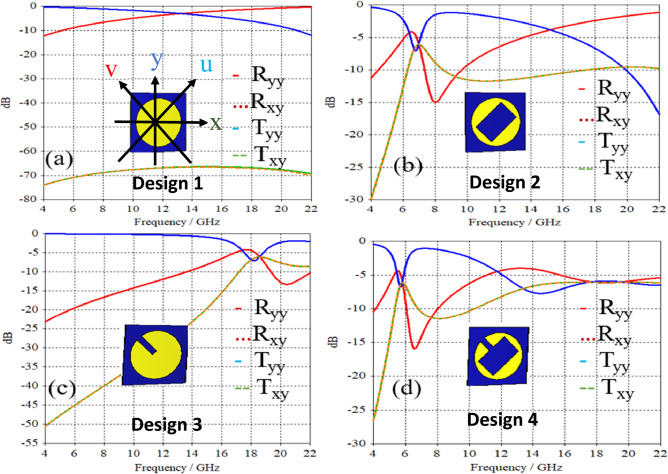
(**a**) No beam splitting (**b**) beam splitting around 6 GHz (**c**) beam splitting around 18.5 GHz (**d**) beam splitting at multiple frequency bands.

It is essential to mention that a well-thought comprehensive parametric analysis and optimization are performed to reach out the final design. The operating resonance frequency is calculated using the following standard equation.

1 $$C = n\lambda_{eff} = \frac{nc}{{\lambda \sqrt {\varepsilon_{reff} } }}$$

where, C is circumference of the circle or the largest dimension of the patch, $\varepsilon_{reff}$ is the effective permittivity of the dielectric substrate. To achieve resonance “n” should be considered 1,3,5 and so on. Here, the proposed FSS works at 5.87 GHz (mode 1, n = 1), and 17.95 GHz (Mode 3, n = 3)^18^. The beam splitting operation with polarization transformation functionality specifically depends on the anisotropy and geometrical configuration of the unit cell structure^19,20^. To reach such a unique geometry that can offer beam splitting operation at multiple frequency bands, a step-by-step geometric evolution of the FSS structure has been carried out as shown in Fig. 2.

Design 1: In the first step, the isotropic circle patch is designed on a substrate with no ground plane to ensure both reflection and transmission at the same time. It can be seen from the simulation results shown in Fig. 2a, both transmission and reflections are obtained, however, no beam splitting occur as the co components of reflection and transmission remains high while cross components are negligible.

Design 2: In the second phase, we implemented anisotropy along the x- and y-axes and established mirror symmetry at the v-axis by etching the rectangular patch from the circular one and tilting it at 45 degrees. As shown in Fig. 2b, the structure now demonstrates beam-splitting capabilities in the vicinity of the 6.3 GHz frequency band.

Design 3: Likewise, in the third phase, anisotropy and mirror symmetry were introduced by etching the metallic strip from a circular patch and rotating it by 45 degrees. The beam splitting operation in the K-band range is evident from Fig. 2c.

Design 4: In order to augment the anisotropy within the structure and achieve multi-band functionality, we carried out etching on both the metallic strip and the rectangular patch (combining aspects of designs 2 and 3) from the circular patch in the ultimate phase. This resulted in a beam splitting operation at two distinct frequency bands, as depicted in Fig. 2d.

### The performance

#### Beam splitting operation

**Figure 3:**
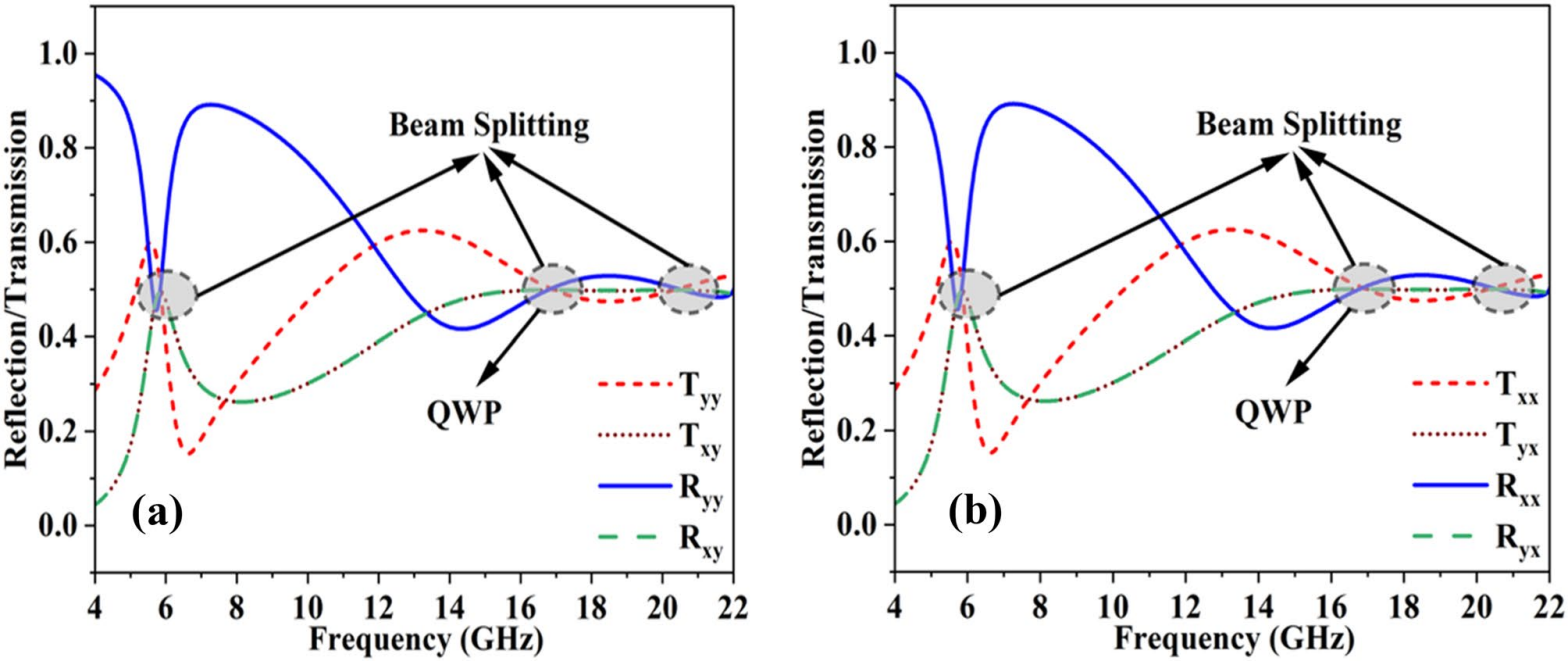
Beam splitting operation when incident wave is (**a**) y-polarized (**b**) x-polarized.

If the x-polarized field is impinging on the proposed structure, then the cross- and co-components of the reflected and transmitted electromagnetic (EM) waves can be seen in Fig. 3. The cross- and co-components of the reflection coefficient are denoted by (R_yx_) and (R_xx_), respectively. Similarly, the cross- and co-components of the transmitting wave are represented by (T_yx_) and (T_xx_). To achieve perfect beam splitting, the co- and cross-components of both reflected and transmitted waves must achieve a 0.5 value. This 0.5 value for both reflection and transmission coefficients ensures the 50% reflection (R^2^_xx_ + R^2^_yx_ = 0.5) and 50% transmission (T^2^_xx_ + T^2^_yx_ = 0.5). When incident waves interact with the proposed FSS, the transmission [(T^2^_xx_ = 0.238) + (T^2^_yx_ = 0.238) = 0.48] and reflection coefficients [(R^2^_xx_ = 0.246) + (R^2^_yx_ = 0.244) = 0.49] of these waves acquire almost an equal magnitude value of 0.5 at three different frequencies of 5.87, 17.95 and 20.2 GHz, as demonstrated in Fig. 3. Furthermore, to verify the loss in the structure, the absorption value is calculated through the absorption formula 1 − R^2^_xx_ − R^2^_yx_ − T^2^_xx_ − T^2^_yx_ = 0. Only 3% of the power is absorbed; the remaining 97% of the power contributes to the beam splitting operation 1-(0.48) -(0.49) = 0.03. At all three resonances of 5.87, 17.95, and 20.2 GHz, the structure almost behaves like a lossless structure.

#### Quarter-wave plate operation

The reflected and transmitted electric fields are composed of both polarization components (x and y), regardless of whether the impinging E-field has a single component. The transmission coefficient (Tij) is a fraction of the transmitted and incident E-fields with polarizations i and j, respectively. Likewise, (Rij) is the reflection coefficient, which can be characterized as a proportion of the reflected and incident E-fields with polarizations i and j, respectively. For linear and circularly polarized EM-waves, the linearly polarized components are marked as x and y, and the circularly polarized components are labelled as " + " (RHCP) and "-" (LHCP), respectively. The Jones reflection coefficient matrix used for numerical representation of cross- and co- on a Cartesian basis is given below:

2 $$T = \left( {\begin{array}{*{20}c} {T_{xx} } & {T_{xy} } \\ {T_{yx} } & {T_{yy} } \\ \end{array} } \right), R = \left( {\begin{array}{*{20}c} {R_{xx} } & {R_{xy} } \\ {R_{yx} } & {R_{yy} } \\ \end{array} } \right)$$

The Jones matrix shows the conversion of impinging linearly polarized E-fields to circularly polarized EM-waves in reflection and transmission mode through the following relation:

3 $$\left( {\begin{array}{*{20}c} {E_{ + r} } \\ {E_{ - r} } \\ \end{array} } \right) = \left( {\begin{array}{*{20}c} {R_{ + x} R_{ + y} } \\ {R_{ - x} R_{ - y} } \\ \end{array} } \right)\left( {\begin{array}{*{20}c} {E_{xi} } \\ {E_{yi} } \\ \end{array} } \right){ } = R_{cl} \left( {\begin{array}{*{20}c} {E_{xi} } \\ {E_{yi} } \\ \end{array} } \right)$$

4 $$\left( {\begin{array}{*{20}c} {E_{ + r} } \\ {E_{ - r} } \\ \end{array} } \right) = \left( {\begin{array}{*{20}c} {E_{x} r + jE_{yr} } \\ {E_{x} r - jE_{yr} } \\ \end{array} } \right) = \frac{1}{\sqrt 2 }\left( {\begin{array}{*{20}c} {R_{xx} + iR_{yx} R_{xy} + iR_{yy} } \\ {R_{xx} - iR_{yx} R_{xy} - iR_{yy} } \\ \end{array} } \right)\left( {\begin{array}{*{20}c} {E_{xi} } \\ {E_{yi} } \\ \end{array} } \right)$$

Similarly, the Jones matrix for transmission coefficient, to transform the linearly polarized E-fields into circularly polarized EM waves, can be modified by replacing the reflection coefficient values of Eqs. (3–4) with transmission coefficient values.

5 $$\left( {\begin{array}{*{20}c} {E_{ + t} } \\ {E_{ - t} } \\ \end{array} } \right) = \left( {\begin{array}{*{20}c} {T_{ + x} T_{ + y} } \\ {T_{ - x} T_{ - y} } \\ \end{array} } \right)\left( {\begin{array}{*{20}c} {E_{xi} } \\ {E_{yi} } \\ \end{array} } \right){ } = T_{cl} \left( {\begin{array}{*{20}c} {E_{xi} } \\ {E_{yi} } \\ \end{array} } \right)$$

6 $$\left( {\begin{array}{*{20}c} {E_{ + t} } \\ {E_{ - t} } \\ \end{array} } \right) = \left( {\begin{array}{*{20}c} {E_{x} t + jE_{yt} } \\ {E_{x} t - jE_{yt} } \\ \end{array} } \right) = \frac{1}{\sqrt 2 }\left( {\begin{array}{*{20}c} {T_{xx} + iT_{yx} T_{xy} + iT_{yy} } \\ {T_{xx} - iT_{yx} T_{xy} - iT_{yy} } \\ \end{array} } \right)\left( {\begin{array}{*{20}c} {E_{xi} } \\ {E_{yi} } \\ \end{array} } \right)$$

Here E_xi_ and E_yi_ are the incident E-field components in the x and y directions. Similarly, E_+r_, E_-r_, E_+t_, E_-t_ are RHCP and LHCP transmitted/reflected E-field components, respectively.

**Figure 4:**
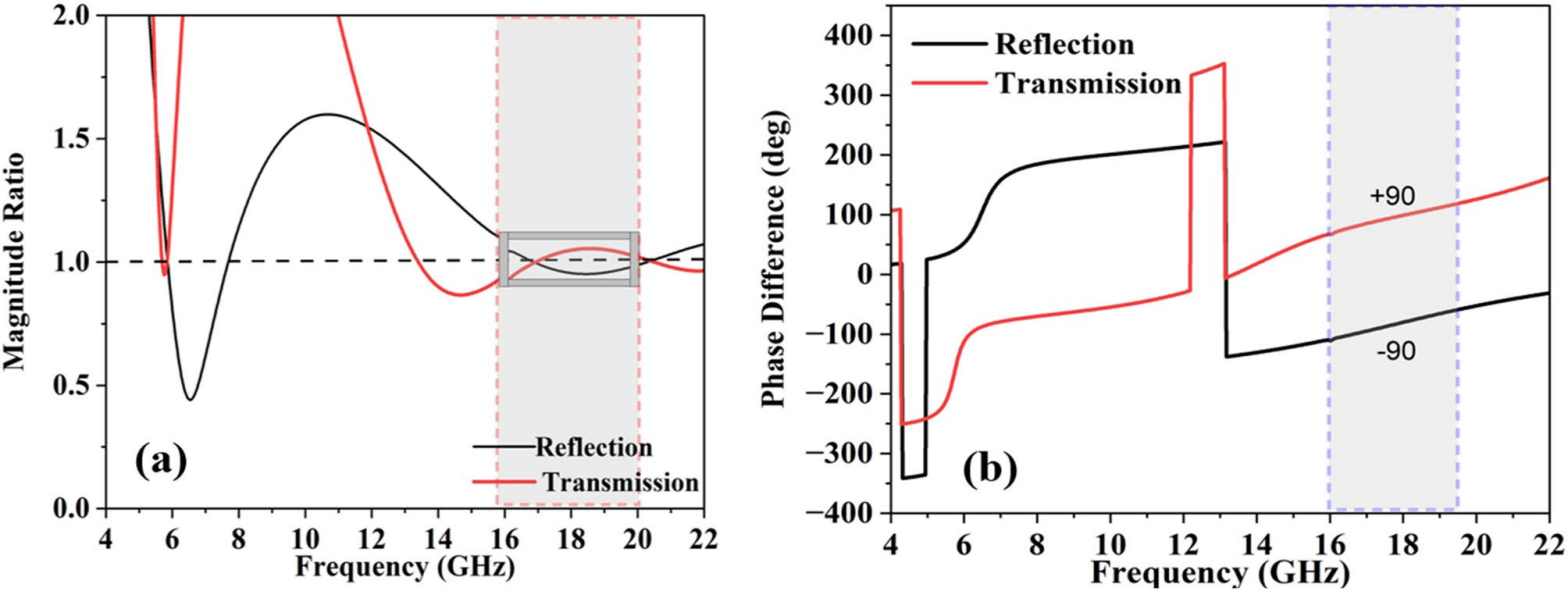
(**a**) Magnitude ratio (**b**) phase difference.

A birefringent structure can control the polarization state; however, it is only possible when one component of the incident wave is delayed by the second component and produces some phase difference between them. If the magnitude ratio between the two linearly polarized components is 1 and the phase difference is 90^°^, then the structure behaves as a QWP. The acceptable criterion of magnitude and phase for good QWP performance operations is 1 ± 0.15 and 90^°^ ± 15^°^, respectively. As shown in Fig. 4a, b, the proposed FSS fulfils the magnitude and phase criteria in the 16.5–18.1 GHz frequency band. The magnitude ratio of 1 ± 0.15 is observed in the wide frequency ranges of 5.8–6.2 GHz and 15.4–22 GHz. However, the 90^°^ ± 15^°^ phase is achieved at the frequency band of 16.5–18.1 GHz. Hence, the power beam splitting operation is achieved in the frequency ranges of 5.8–6.2 GHz and 15.4–22 GHz, while the twofold operation is achieved in the frequency band of 16.5–18.1 GHz.

#### The axial ratio, the polarization extinction ratio and the ellipticity

**Figure 5:**
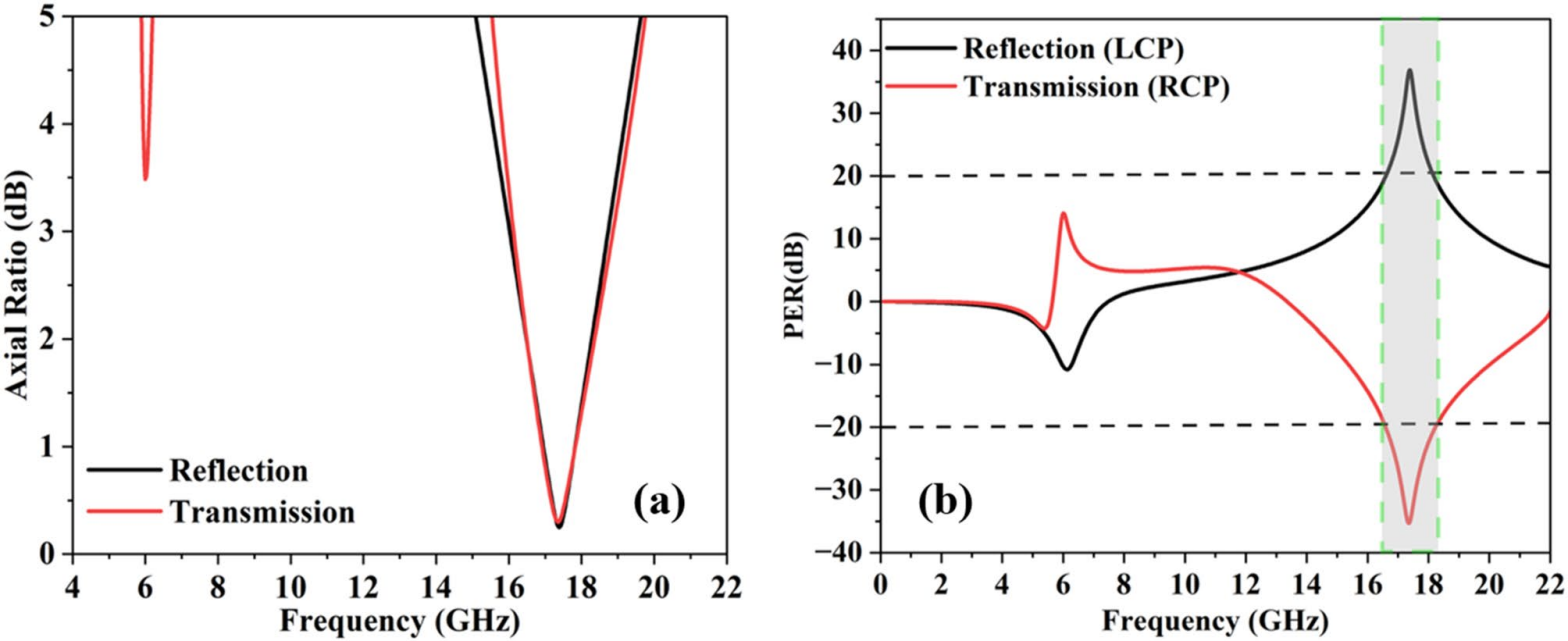
(**a**) Axial ratio (dB), (**b**) polarization extinction ratio (dB).

To counter-verify the circular polarization conversion, the axial ratio (dB) is calculated by the following equation:

7 $$AR = \left( {\frac{{\left| {R_{yy} } \right|^{2} + \left| {R_{yy} } \right|^{2} + \sqrt b }}{{\left| {R_{yy} } \right|^{2} + \left| {R_{xy} } \right|^{2} - \sqrt b }}} \right)^{1/2}$$

where b = $\left[ {\left| {R_{yy} } \right|^{2} + \left| {R_{xy} } \right|^{2} + 2\left| {R_{yy} } \right|^{2} \left| {R_{xy} } \right|^{2} {\text{cos}}\left( {2\Delta \varphi_{xy} } \right)} \right]$ and $\Delta \varphi_{xy}$ is the phase difference. The acceptable criterion for a circularly polarized wave is AR $\le 3{ }dB$^18^. As seen from Fig. 5a, the 3 dB AR is provided in the frequency range of 16.5–18.2 GHz.

The handedness of the CP-wave is also important for various applications in the microwave regime. The best way to analyze the handedness of the CP-wave is through the polarization extension ratio (PER).

8 $$PER = 20log_{10} \left( {\frac{{\left| {R_{yx} } \right|^{2} }}{{\left| {R_{yy} } \right|^{2} }}} \right)$$

The reflected or transmitted wave is a left-handed circularly polarized wave (LCP) if the polarization extinction ratio (PER) is 20 dB or above. On the other hand, the reflected or transmitted wave is a right-handed circularly polarized wave (RCP) if the PER value is below -20 dB^21^. The numerical simulation of Fig. 5b depicts that the reflected wave is an LCP-wave in the frequency band of 16.5–18.2 GHz, while the transmitted wave is an RCP-wave in the same frequency band, respectively.

To counter verify the rotation and polarization state of the incident EM-wave, the ellipticity $({\upeta }$) of the EM-wave can be calculated from Eq. (9).

9 $${\upeta } = \frac{1}{2}\sin^{ - 1} \left\{ {\frac{2R\sin \varphi }{{1 + R^{2} }}} \right\}$$

where ${ }R = \left| {T_{xy} } \right|/\left| {T_{yy} } \right|$ and $\varphi = arg\left( {T_{xy} } \right) - \left( {T_{yy} } \right)$ or $R = \left| {R_{xy} } \right|/\left| {R_{yy} } \right|$ and $\varphi = arg\left( {R_{xy} } \right) - \left( {R_{yy} } \right)$.

**Figure 6:**
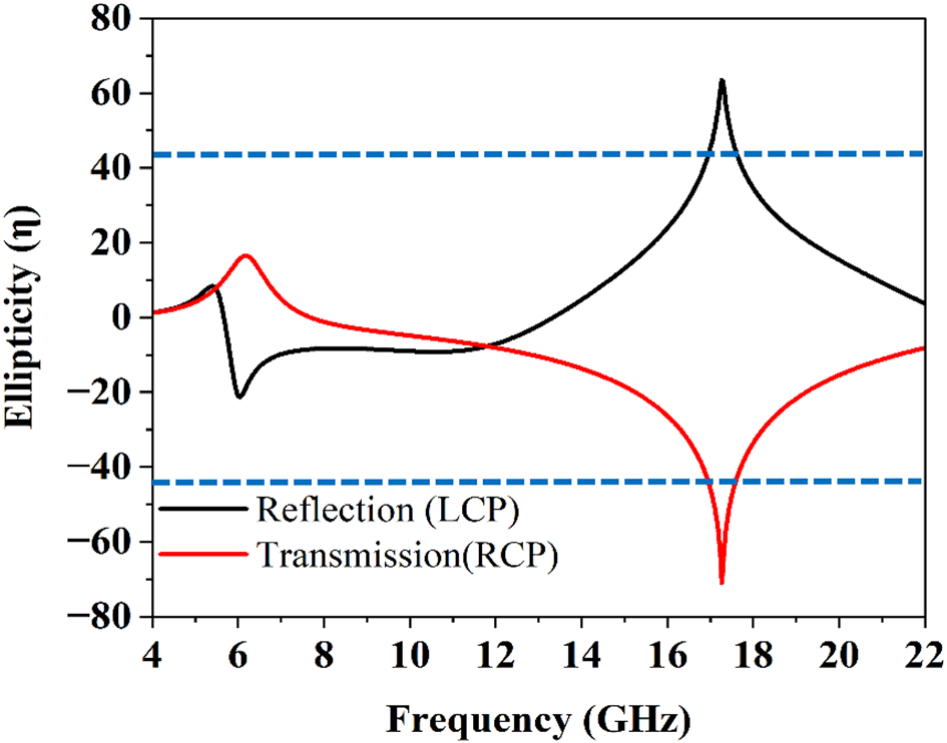
The ellipticity operation.

At the point when η is greater than zero, the wave is left-handed ellipticity polarization; similarly, if η is less than zero right-handed ellipticity polarization is obtained. A pure circular polarization state can be achieved if and only if η is exactly equal to 45^°^^22^. Figure 6 demonstrates that the ellipticity is approaching 45^°^ for both reflected and transmitted waves. The ellipticity criterion is fulfilling for circular polarization state in the required frequency band for both reflected and transmitted waves.

### The underlying physical mechanism of polarization transformation phenomenon

The dual operation, encompassing both simultaneous beam splitting and quarter-wave plate (QWP) functionality, is realized within the frequency range of 16.5–18.2 GHz. It is imperative to analyze the underlying physical mechanism governing the transformation from linear to circular polarization. Therefore, the Jones matrix governing the transmission and reflection coefficients is presented in Eq. (2).

10 $$T = 0.5\left( {\begin{array}{*{20}c} {e^{{ - i\frac{\pi }{4}}} } & {e^{{ - i\frac{\pi }{4}}} } \\ {e^{{ - i\frac{\pi }{4}}} } & {e^{{ - i\frac{\pi }{4}}} } \\ \end{array} } \right),\quad {\text{R}} = 0.5\left( {\begin{array}{*{20}c} {e^{{ - i\frac{\pi }{4}}} } & {e^{{ - i\frac{\pi }{4}}} } \\ {e^{{ - i\frac{\pi }{4}}} } & {e^{{ - i\frac{\pi }{4}}} } \\ \end{array} } \right)$$

**Figure 7:**
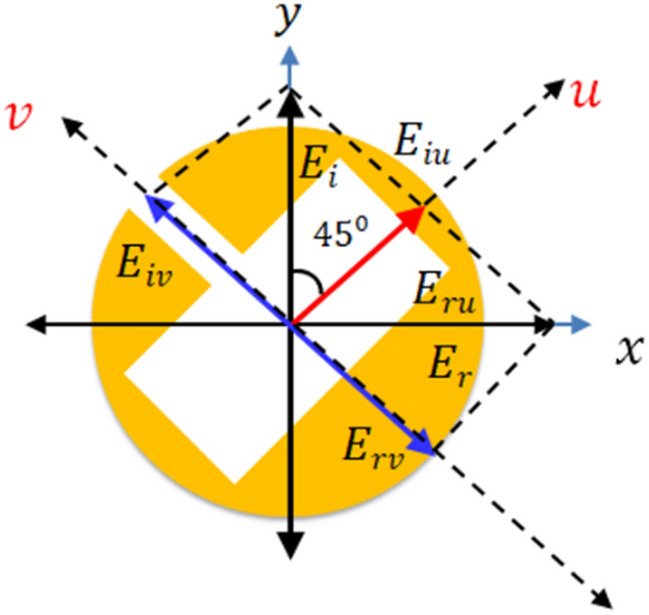
(**a**) UV-analysis of the proposed FSS for simultaneous beam splitting and QWP operation.

Here T/R is the transmission/reflection matrix, $u = \left( {1 1} \right)^{2}$ and $v = \left( {1 - 1} \right)^{2}$ with the eigenvalues of $\frac{\sqrt 2 }{2}$ and $\frac{\sqrt 2 }{2}e^{{i\frac{\pi }{4}}}$ , these are linearly independent eigenvectors, respectively. The u- and v-eigenvectors are oriented at ± 45° to the y-axis and x-axis, as depicted in Fig. 7. The eigenvalues show that eigenpolarization can be reflected with 50% power by keeping the polarization state unaltered. Furthermore, the phase of the FSS is 0° for u-polarization and -90° for v-polarization. Similarly, the reflected component can be replaced by transmitted electric filed to analyse the response in the transmission mode.

**Figure 8:**
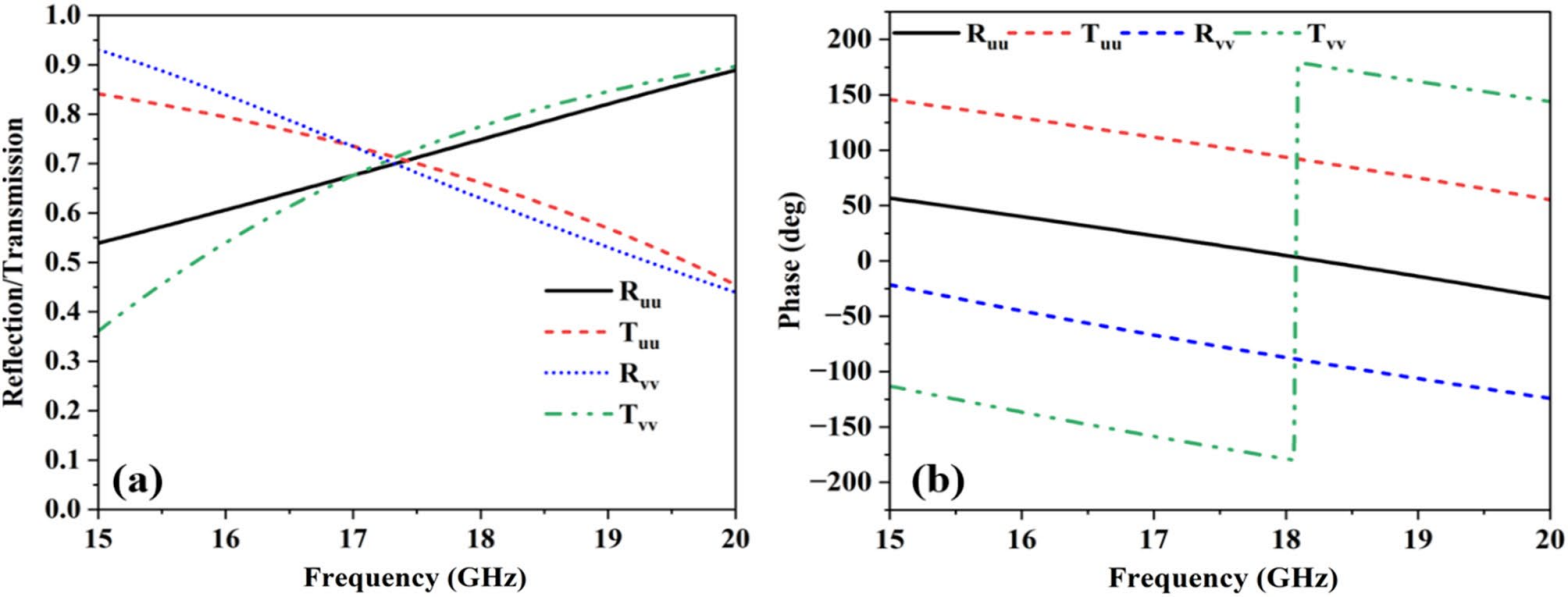
(**a**) Magnitude for UV-incident wave (**b**) phase for UV-incident wave.

To validate the aforementioned analysis, numerical simulations are performed for both u- and v- polarization. The magnitude and phase of the transmitted and reflected fields are demonstrated in Fig. 8a and b, respectively. It is clear from Fig. 8a, b that the magnitude value obtained for the transmission coefficients for a co-polarized wave has achieved the same value of (0.707) for both u- and v-polarization, and the phase obtained from them is 0° and -90°, respectively. The same is the case with reflection coefficients. Since linearly polarized fields (x and y) are broken into their orthogonal components (u and v), so, QWP operation is only possible when u- and v-components of transmitted and reflected waves attain the same magnitude and 90° phase difference. As the proposed FSS fulfils the magnitude and phase requirement in the frequency range of 16.5–18.2 GHz. Hence, for eigenpolarizations, the proposed structure acts as a 1:1 beam splitter.

**Figure 9:**
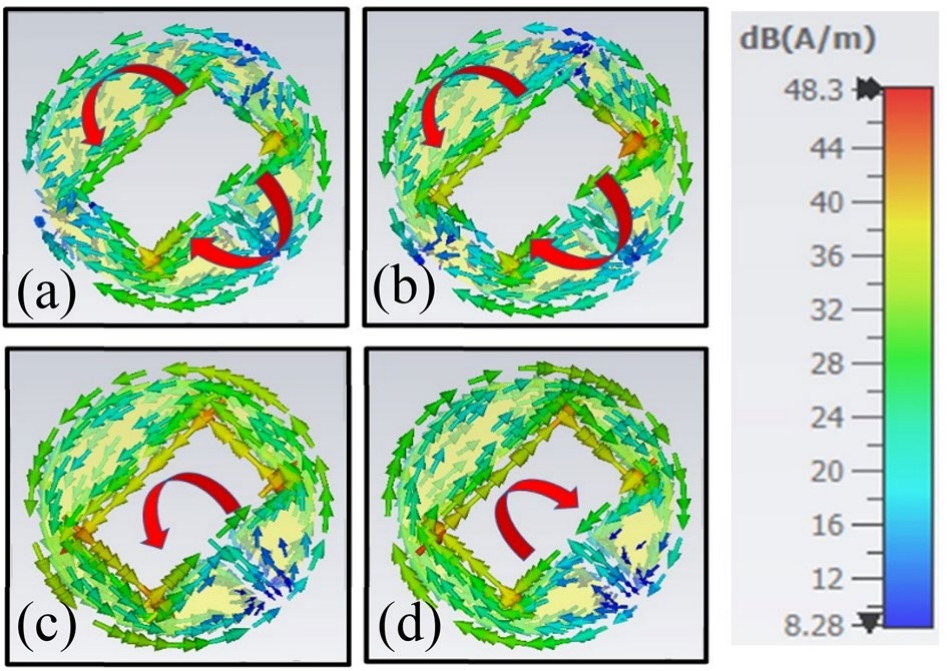
Surface currents distribution (**a**) 5.87 GHz (reflection) (**b**) 5.87 GHz (transmission) (**c**) 17.95 GHz (reflection) (**d**) 17.95 GHz (transmission).

To get more physical insight, the current distribution analysis has been performed at resonance frequencies of 5.87 and 17.95 GHz. The rotation of current on the surface of the structure determines the polarization state of the EM wave. The wave is LCP if the rotation of the current flow is anticlockwise, while it is RCP if it is in a clockwise direction. The current distribution remains random, or it is cancelled out if no polarization transformation occurs. It is clear from Fig. 9a and b that no polarization conversion takes place at 5.87 GHz as the current flow is in the same direction during reflection and transmission. On the other hand, as can be seen from Fig. 9c and d, the rotation of current flow is anti-clockwise upon reflection and clockwise upon transmission at 17.95 GHz. Thus, left-handed linear to circular and right-handed linear to circular polarization transformations occur upon reflection and transmission of the wave, respectively.

### Angular stability and bending analysis

**Figure 10:**
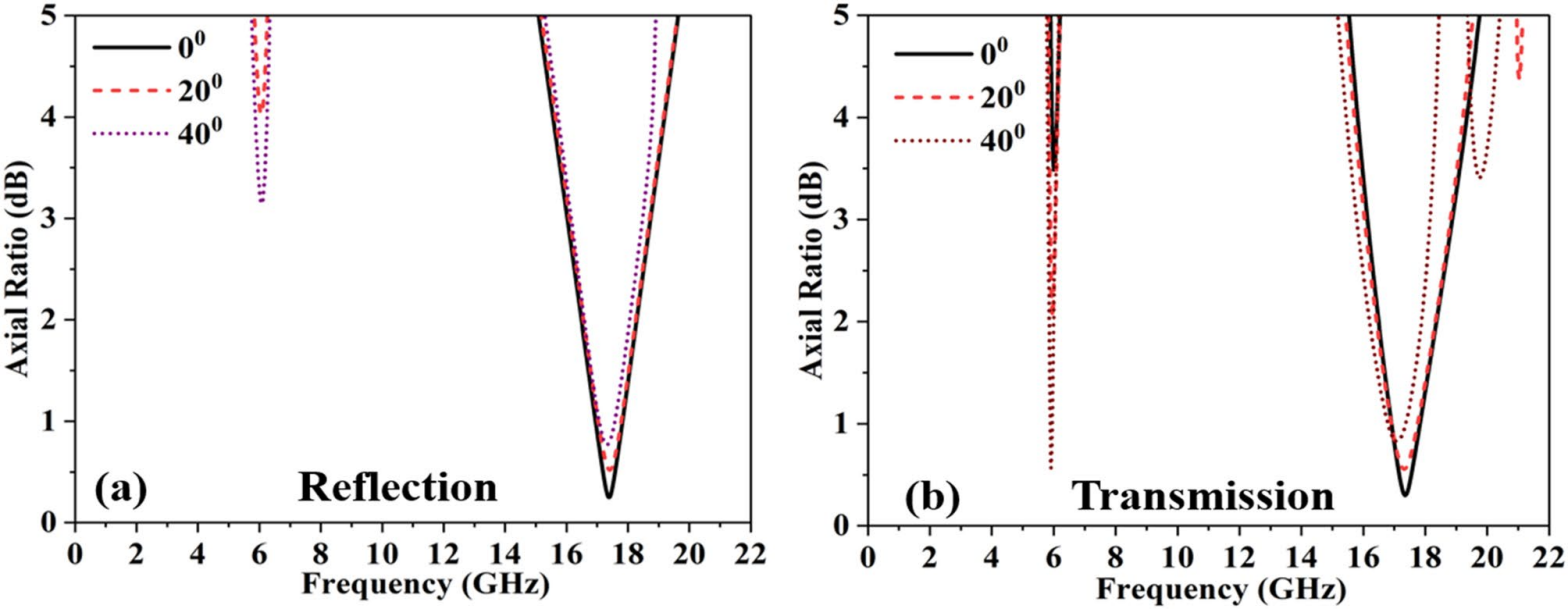
Angular stability (**a**) axial ratio in reflection mode. (**b**) Axial ratio in transmission mode.

Several practical applications necessitate consistent behavior of frequency selective surfaces under variations in the incident angle. Typically, this angular stability is contingent on the dielectric constant of the substrate. Achieving high stability in the structure demands a higher dielectric value, but this can detrimentally impact the structure's bandwidth^23^. An alternative approach to attain angular stability involves incorporating vias within the substrate, strategically placed among unit cells to mitigate interference stemming from multiple reflections^24^. However, this technique is not viable for the suggested structure, given the flexible substrate and absence of a ground plane. Consequently, the sole method to ensure angular stability in the structure is through the distinctive physical configuration of the unit cell and its sub-wavelength dimensions^25^. As depicted in Fig. 10a and b, the axial ratio remains stable for both reflected and transmitted waves even under oblique incidence up to 40 degrees. The emergence of additional resonances is attributed to spatial dispersion, which occurs in periodic structures under oblique incidences^26^. In the case of the proposed beam-splitting Frequency Selective Surface (FSS), the electrical length of the period expands with higher incidence angles, leading to the manifestation of spatial dispersion.

**Figure 11:**
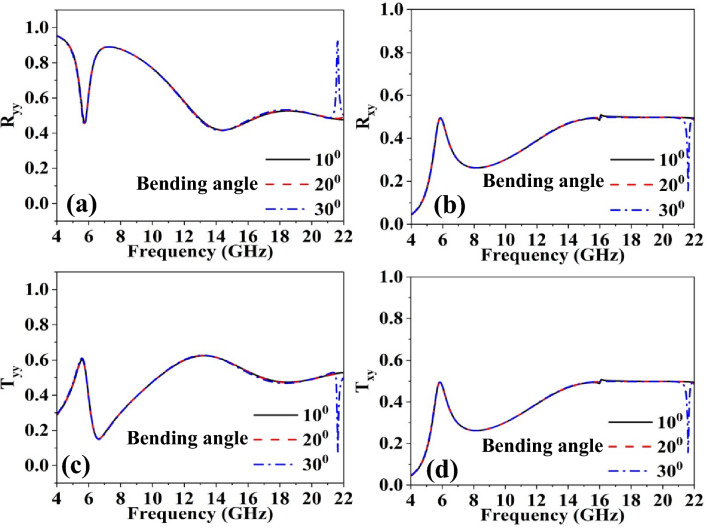
Beam splitting operation versus different angles of curvature.

**Figure 12:**
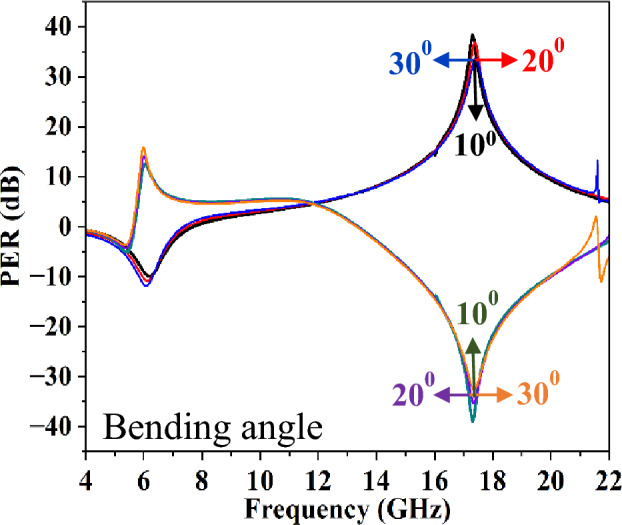
Polarization extinction ratio (PER) versus different angles of curvature.

Conformal devices also require a stable performance across various bending in the curvature. Hence, it is imperative to investigate the impact of bending the structure on its performance at different angles in order to assess its influence on polarization conversion efficiency and beam-splitting functionality. The full-wave analysis have been carried out versus different bending angles as depicted in Figs. 11 and 12. It has been observed that the FSS's performance in terms of beam splitting and polarization conversion remains largely unaffected up to 30° of bending angle, respectively. However, additional resonances in the response are emerged beyond 20.5 GHz.

### The equivalent circuit model

**Figure 13:**
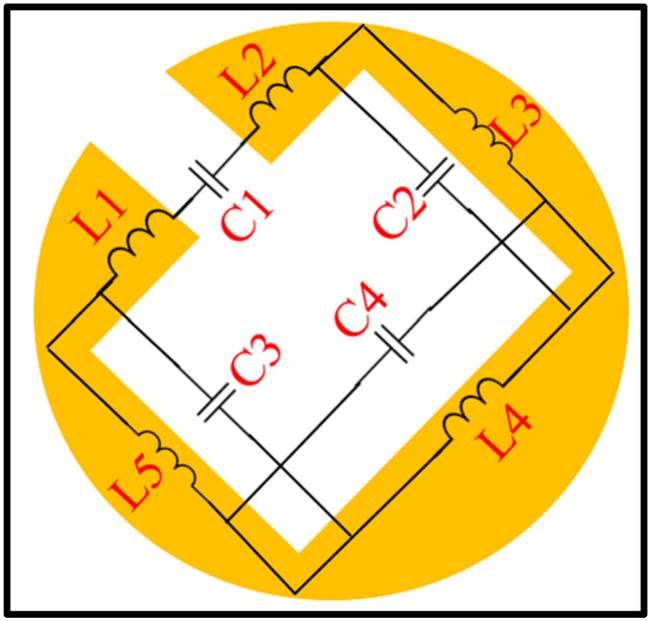
The equivalent circuit model of the proposed FSS.

**Figure 14:**
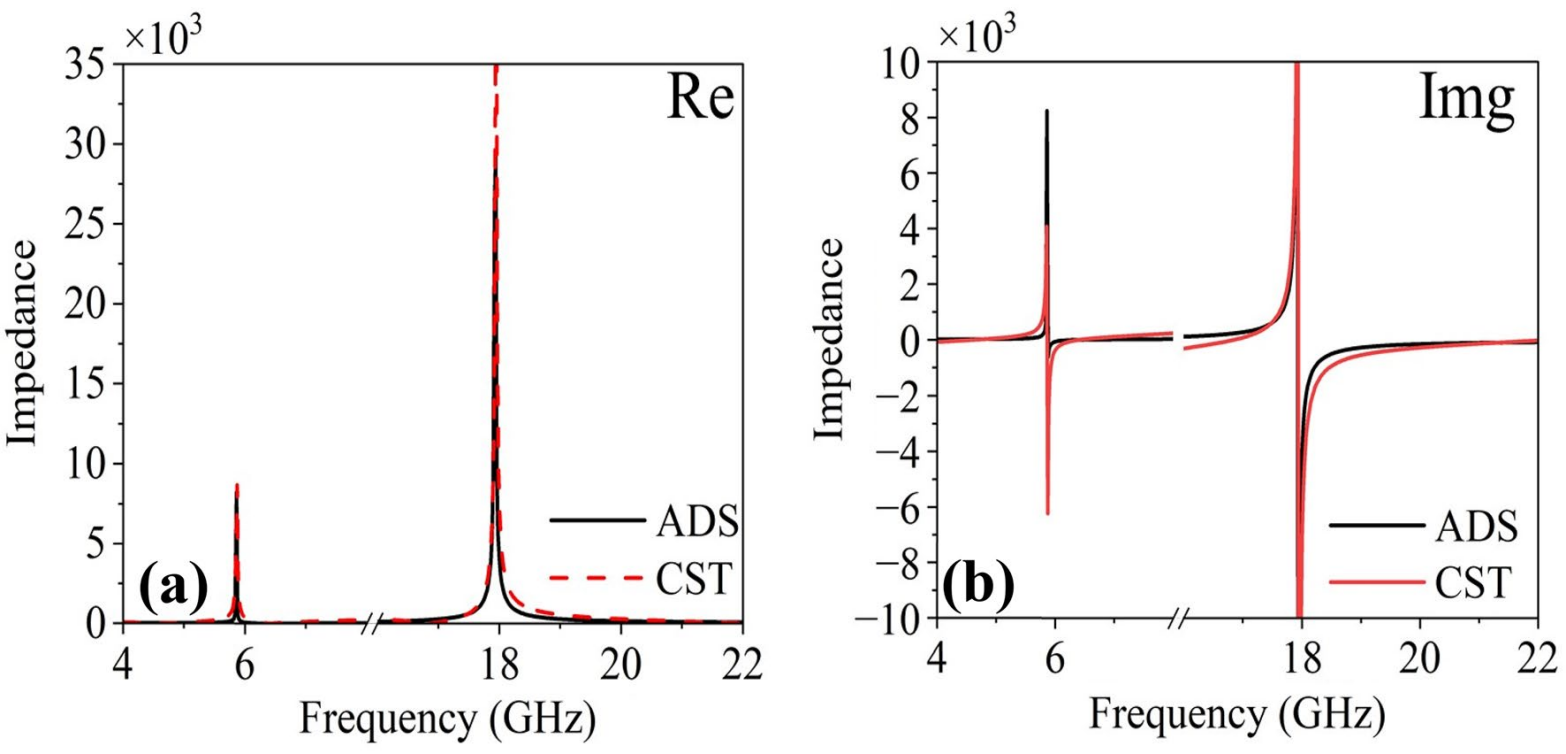
The impedance (**a**) real part (**b**) imaginary part.

The equivalent circuit model of the proposed FSS is shown in Fig. 13. The inductance (as a function of strip lengths and widths) and capacitance (as a function of split gap) are represented by L and C, respectively. The impedance of the proposed FSS is retrieved from full-wave simulations using CST microwave studio, and for its verification, the LC model is simulated in ADS (Advanced Design System) software. It can be seen from Fig. 14a and b, the real and imaginary parts of the impedance match with each other. The inductance and capacitance values of the proposed FSS are C1 = 0.64 pF, C2 = 0.95 pF, C3 = 0.27 pF, C4 = 0.49 pF, L1 = 0.1 nH, L2 = 1.099 nH, L3 = 0.48 nH, L4 = 0.468 nH and L5 = 1.907 nH. In order to tune the resonance frequency (i.e., $f_{r} = \frac{1}{{2\pi \sqrt {LC } }}$), the inductance (as a function of strip lengths and widths) and capacitance (gap) of the structure can be varied by altering the size and shape of the structure. The resonant frequency will move towards the higher side by decreasing the value of the capacitance (C1-C4) or inductance (L1-L5) and vice versa.

Table 1 provides a comprehensive comparison with recently published beam splitters. The proposed FSS clearly outperforms other beam splitters in several key aspects. Firstly, it is conformal and operates effectively in both transmission and reflection modes. Secondly, it demonstrates multiple operations across multiple frequency bands.

**Table 1:** Comparison with other beam splitters.

Ref	BM without QWP	BM with QWP	Conformal	Angular Stability	Both reflection and transmission modes
^6^	THz	N/A	No	No	No
^11^	N/A	11.65–11,395 GHz	Yes	No	NO
^15^	N/A	9 GHz	No	No	NO
^16^	THz	N/A	No	No	NO
^17^	N/A	4.2 GHz	No	No	NO
This work	5.8–6.2 GHz, 18.5–22 GHz	16.5–18.2 GHz	Yes	40^°^	Yes

BM: Beam splitting; QWP = Quarter-wave plate.

## Experimentation

**Figure 15:**
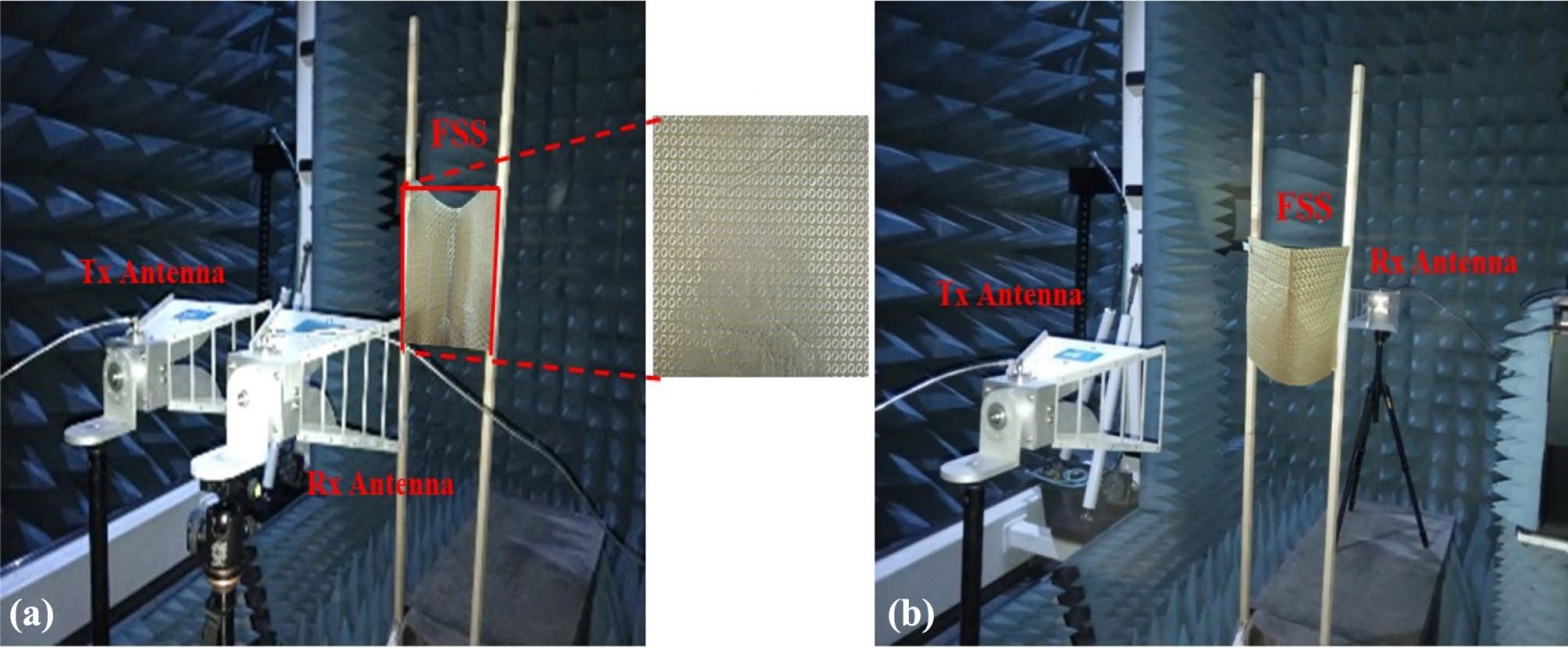
Measurement setup (**a**) reflection mode (**b**) transmissions mode.

**Figure 16:**
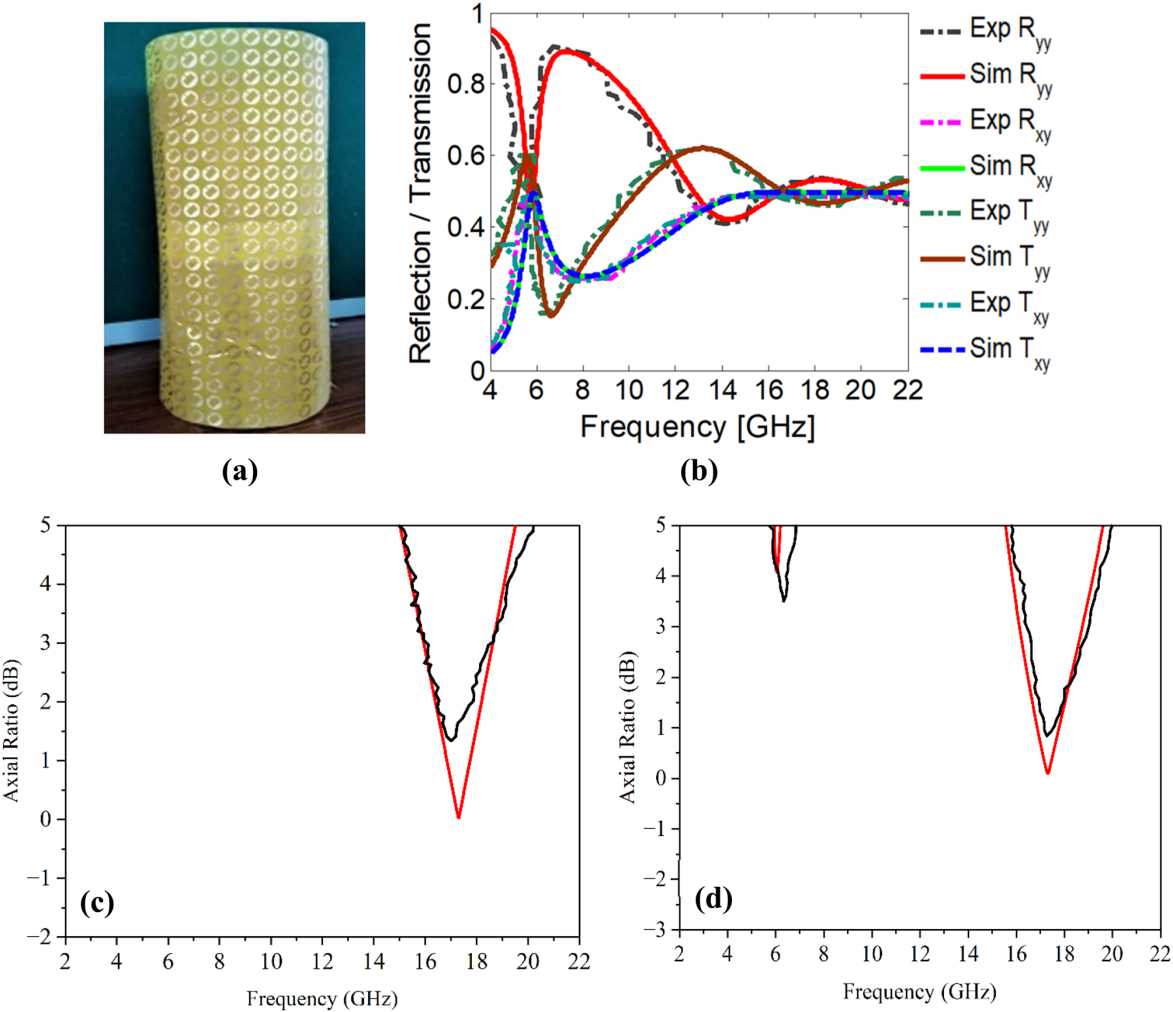
(**a**) The fabricated flexible prototype (**b**) reflection/transmission results (**c**) axial ratio reflection (**d**) axial ratio transmission.

Measurements have been carried out to experimentally validate the full-wave simulations. The designed conformal FSS is fabricated on a 330 × 330 × 0.06 mm^3^ polyimide substrate as shown in Fig. 16a. The fabricated prototype consists of 30 × 30-unit cells printed using standard printed circuit board techniques. For experimentation, four wideband horn antennas with an operating frequency band of 1–18 GHz (two horns) and 18–26 GHz (two horns) are utilized for illuminating the surface and then receiving the reflected and transmitting waves. For reflection measurements, the receiving and transmitting antennas are placed on the same side as shown in Fig. 15a. Similarly, for transmission measurements, the receiving and transmitting antennas are placed on the opposite side to each other facing the FSS as shown in Fig.15b. To cater the environmental effects, measurements are performed for air and copper plates and later subtracted from obtained experimental results FSS. As shown in Fig. 16b, the simulation and measurement results of the FSS for both reflection and transmission are in agreement and exhibit beam splitting operation. Similarly, Fig. 16c and d show the axial ratio in both reflection and transmission modes. The small variations between measurements and simulations are caused by imperfections in the prototyping, dielectric tolerance, finite sample size, and limitations of the measurement equipment.

## Conclusion

This paper introduces an ultrathin, flexible, and conformal Frequency Selective Surface (FSS)-based beam splitter. The proposed structure achieves a balanced beam-splitting operation (50% reflection and 50% transmission) across two frequency bands, and simultaneous beam splitting with quarter-wave operation in the 16.5–18.2 GHz range. Additionally, the FSS exhibits angular stability up to 40 degrees against oblique incidences. Conformal beam splitting FSSs optimize space utilization and efficiently integrate with complex surfaces, including curved or irregularly shaped objects. These attributes position the proposed conformal beam-splitting FSS as a promising candidate for various applications, such as interferometers, spectrometers, quantum optics, optical communication, and RIS-assisted network systems.

## Data Availability

The datasets generated during and/or analyzed during the current study are available from the corresponding author on reasonable request.
